# The Joint Effects of Hubris, Growth Aspirations, and Entrepreneurial Phases for Innovative Behavior

**DOI:** 10.3389/fpsyg.2022.831058

**Published:** 2022-02-25

**Authors:** Carlos Poblete

**Affiliations:** School of Business and Economics, Universidad del Desarrollo, Santiago, Chile

**Keywords:** hubris, growth aspirations, innovation, entrepreneurial process, entrepreneurial ambition

## Abstract

Innovation is often seen as essential for ventures to succeed. High business failure rates in entrepreneurship, however, suggest that innovations are frequently driven by entrepreneurs blinded by overconfidence. Thus, anticipating when and why entrepreneurs will be motivated to innovate is fundamental for entrepreneurial success. Using a large sample obtained from population surveys conducted in 77 countries, this study analyzes the variables that are significantly associated with innovative behaviors. The research tests a model proposing that the joint effects of hubris, growth aspirations, and an entrepreneur’s level of entrepreneurial experience have a crucial impact on innovative endeavors. It finds that hubris is significantly related to entrepreneurs’ growth aspirations and that ambition, in turn, is positively related to innovative behaviors. In addition, the study finds that both relationships are moderated by the level of entrepreneurial experience. These findings highlight the need to wise up amateur entrepreneurs before they embark on innovative endeavors.

## Introduction

Extensive statistical evidence shows that entrepreneurship, as an economic activity, is characterized for having high rates of business failure (Headd, 2001; Hayward et al., 2006). For example, the Failure Institute state that 75% of new firms in developing economies do not survive more than 2 years. Shane (2009) noted that the correlation across industries between start-up rates and failure rates is 0.77. Headd (2003) observed that 34% of new ventures did not survive the first 2 years, 50% did not survive 4 years, and 60% did not survive 6 years. Furthermore, studies reveal that, on average, 9 out of 10 new businesses close during their first year (e.g., Phillips and Kirchhoff, 1989). In a similar vein (Dunne et al., 1989), analyzing the manufacturing sector, observed that 62–80% of firms existed the market after a period of 5–10 years, with most exits being failures. Despite the above, many of those who engage in entrepreneurial activities are driven by the belief that they can overcome great odds and achieve success (Zhang and Cueto, 2017). This phenomenon—which Scott Shane refers to as “the myth of entrepreneurship” (Shane, 2008) —is strongly related to individuals who present tenacity, excessive pride, and arrogance (Shane, 2009).

Theoretical studies propose that individuals’ socially constructed confidence affects how they interpret information about their ventures (Hayward et al., 2006, 2010). Literature also suggests that when people act on fictional thinking as it was reality, they often get hurt and also harm those around them (Shane, 2008; Trevelyan, 2008). This, however, may not be entirely true, as shown by the conflicting views and evidence on whether the resulting outcomes are positive or negative. For instance, several studies observed that overly confident entrepreneurs frequently err when deciding how to optimally allocate resources (e.g., De Meza et al., 1996; Shane, 2009). It has also been suggested that the typical entrepreneur is bad at selecting industries, as they commonly choose the easiest, rather than the best industries for starting up a business (Johnson, 2004). While not every business closure should be seen as a failure, entrepreneurs tend to overestimate their abilities (Cassar, 2010; Coelho, 2010; Mueller and Shepherd, 2016), and this in turn is related with some of the observed discontinuance rates of entrepreneurial activity. On the other hand, extensive evidence suggests that positive beliefs can contribute to the achievement of myriad outcomes. For instance, passion (Cardon et al., 2009, 2017), courage (Miller and Le Breton-Miller, 2017; Bockorny and Youssef-Morgan, 2019), and psychological capital (Jensen and Luthans, 2006; Rauch and Frese, 2007) are positively related to venture performance (Hmieleski and Carr, 2008), authentic leadership (Jensen and Luthans, 2006), and wellbeing (Hmieleski and Carr, 2007).

Inspired by this paradoxical evidence, this paper aims to understand the role of entrepreneurs’ cognitive processes on firm-level innovation. Specifically, this study adopts an integrative approach based on social cognitive theory (SCT) and the hubris theory of entrepreneurship (HTE) to explain how some cognitive processes (i.e., hubris, comprising founder’s confidence in knowledge, predictions, and personal abilities) of the entrepreneurs’ managerial decisions (i.e., enacting innovative endeavors) change across the entrepreneurial process. Using a sample of 104.564 entrepreneurs, findings underscore the moderating role of the entrepreneurial process on the relationship between hubris and growth aspirations, and between growth aspirations and innovation. It finds that both relationships become weaker as entrepreneurs progress through the entrepreneurial process.

This study offers three key contributions to the ongoing academic debate on this topic. First, it examines the applicability of complementing SCT and HTE for explaining when, how and why hubris has a direct—and indirect—impact on innovative endeavors through growth aspirations. By doing so, this research confers theoretical grounds to support existing empirical evidence, helping improve the understanding of the role of hubris in entrepreneurs’ characteristics (e.g., motives, goals, values) and firm’s outcomes (e.g., innovative outcomes). It is anticipated that entrepreneurs’ initial cognitive settings have a relative influence on a firm’s outcomes. This, via the construction of a strong intrinsic motivation that leads entrepreneurs to make risky decisions and to integrate this as a core aspect of their business strategies. Thus, the present research contributes to continuing efforts to elucidate how hubristic entrepreneurs set their ambitions for growth and the subsequent impact that this has on multiple innovative endeavors. Second, while the existing literature provides consistent findings of the role of experience in entrepreneurship, it fails to directly address how the entrepreneurial process provides reference points through which the relative influence of initial cognitive settings on firm outcomes change as entrepreneurs advance through the phases of the entrepreneurial venture. The present research addresses this possibility by investigating the moderating role of the entrepreneurial stages in how hubristic entrepreneurs set their ambitions for growth. While recent studies emphasize the benefits and downsides of hubris among entrepreneurs, limited focus has been placed on how its influence might vary across the different entrepreneurial phases. Third, similar to other studies (Anokhin and Wincent, 2012), this research warns of the nuances and complexities of encouraging entrepreneurs to develop innovative ventures, as those most likely to be persuaded are amateurs and naive entrepreneurs (Mitchell et al., 2008; Senyard et al., 2014; Sanchez and Dunning, 2018). These insights can help inform and shape policy initiatives aimed at fostering innovation by highlighting how hubris can act as a precursor to innovative endeavors. The findings call for a serious reconsideration of the premise on which new venture creation support programs are built. From a public policy perspective, stimulating start-up rates to encourage innovative entrepreneurial outcomes may be inadequate. Moreover, public investments of pecuniary and non-pecuniary resources into such programs must be guided by a sound understanding of entrepreneurs’ motivations. In the absence of such information, private and public investments might end up boosting ambitious entrepreneurial ventures that are likely to fail, resulting in substandard outcomes for the local economy.

## Theoretical Underpinning

SCT is based on the principle that personal and situational factors affect individuals’ social behaviors (Bandura, 1977). Social cognition analysis considers the mental representations that are constructed based on people’s current or previous experiences, how these representations are manipulated, the processes through which they influence other aspects of cognition and the decisions and behaviors that result from the application of these processes (Bodenhausen et al., 2003). To do so, SCT adopts an agentic perspective to self-development, adaptation, and change (Bandura, 2005). To be an agent is to intentionally make things happen through one’s actions (Bandura, 2001). Hence, individuals are not only planners and forethinkers but also self-regulators. One of the means through which individuals regulate themselves is through self-efficacy. Self-efficacy is defined as the belief in one’s capability to mobilize the cognitive resources and the actions needed to exercise control over events in our lives (Wood and Bandura, 1989). According to SCT, self-efficacy is a major determinant of people’s choice of activities, how much effort they will expend, and how long they will sustain effort when dealing with stressful situations (Bandura, 1977).

Alongside SCT, HTE (Hayward et al., 2006) is a highly influential theory explaining why some entrepreneurs are particularly prone to starting up new ventures under high rates of business failure. Like SCT, this theory refers to an individual’s belief in her/his own ability to accomplish a goal or outcome. HTE focuses on socially constructed confidence (Bollaert and Petit, 2010), where entrepreneurs’ beliefs about the success of a project lie on their interpretation of their knowledge, skills, and the project’s qualities. However, HTE highlights how overconfidence encourages entrepreneurs not only to start firms but also to pursue challenging growth strategies, often in hostile environments with insufficient resources. Thus, these two theories are complementary in the conception of the individual as being goal-directed and proactively involved in shaping the task environment.

A core element of these two theories is the role of cognitive processes in explaining behavior, either through self-efficacy for SCT or overconfidence for HTE. The nexus between these approaches is underpinned by studies observing that confidence is closely related with self-efficacy (Hayward et al., 2010). Overconfidence refers to self-efficacy that exceeds the individual’s capacity to successfully achieve the task at hand (Douglas, 2009, 2017; Hurst, 2019), where hubris can be conceived as the “dark side” of overconfidence (Hayward et al., 2006). According to Akstinaite and Sadler-Smith (2021), hubris is an extreme manifestation of confidence that is described by preoccupations with success, feelings of excessive pride and self-importance. Thus, hubris can be understood as an exaggerated sense of self-efficacy, and as a defining feature of entrepreneurs’ thinking (Hayward et al., 2006). Methodologically, hubris is linked with individuals’ subjective interpretation of information concerning three separate and independent psychological processes: (1) overconfidence in knowledge, (2) overconfidence in prediction, and (3) overconfidence in personal abilities.

Based on these frameworks, it is anticipated that social behavior is triggered by the individual’s expectations which, in turn, are nurtured by the conviction that they can successfully execute the behavior required to produce the outcomes (Shaver, 2003). As a result, individuals might believe that a particular course of action will produce certain outcomes. However, even if they entertain serious doubts about whether they can perform the necessary activities, such information will not necessarily influence their behavior.

## Hypotheses Development

Prior literature describes hubris as exaggerated self-confidence (Hayward and Hambrick, 1997) that arises when entrepreneurs overestimate the personal wealth they will generate from their ventures (Hayward et al., 2006). Empirical studies observe that entrepreneurs are generally highly confident even though traditional entrepreneurial activity is statistically very likely to fail, and initial plans for a venture are a weak predictor of future performance. This suggests that entrepreneurs are prone to overconfidence both in terms of the risk profile of the opportunities they identify and the initial resource endowments deemed sufficient to pursue them (Hayward et al., 2006). Owen and Davidson (2009) identify three key external factors that contribute to hubris: (1) holding substantial power; (2) minimal constraint on the leader exercising authority; (3) the length of time that leaders remain in power. Considering that these three factors can be parsimoniously featured as characteristics of what occurs in entrepreneurial ventures, it is possible to assume that entrepreneurs are particularly prone to hubris.

While some studies suggest that hubris can be attributed to risk-taking (Craig and Amernic, 2004; Li and Tang, 2010), others propose that it involves a “belief in one’s superior qualities” (Chatterjee and Hambrick, 2007). For example, a classic symptom of hubris is an exaggerated self-belief, bordering on a sense of omnipotence regarding what the individual thinks that he/she can personally achieve. This, in turn, can manifest in contempt for the input of others, with entrepreneurs pursuing strategies out of their inflated sense of confidence and impaired convictions (Kroll et al., 2000; Douglas et al., 2021). In this sense, this hubris is driven by an interpretation of their experiences and is largely unaffected by the experiences of others or the features of the situation—even when considering others’ experiences and situational features could help improve the accuracy of decisions (Ball et al., 1991; Moore and Kim, 2003).

At the firm level, the effect of hubris can be observed on the decision-making, specifically on strategic decision processes, strategic choices, and organizational performance (Simon and Houghton, 2003; Hiller and Hambrick, 2005). Hubristic entrepreneurs may not be inclined toward decision comprehensiveness. Instead, they may be likely to believe that they already possess the required skills, valuable personal insights to understand strategic situations and available alternatives, such that they will not feel the need to exhaustively gather, analyze, and discuss data. Accordingly, hubristic entrepreneurs may hold the conviction that their efforts and expectations lead to favorable firm outcomes (Hayward et al., 2010).

At a personal level, the effects of hubris have been linked with a wide array of personality traits, including locus of control, tolerance for ambiguity, charisma, and risk-propensity (Hayward and Hambrick, 1997). For instance, Hiller and Hambrick (2005) suggested that hubristic individuals have a higher locus of control (Miller, 1983; Urbig and Monsen, 2012; Karabulut, 2016). If so, it is reasonable to consider that hubris may be substantially valuable because it allows hubristic entrepreneurs to create and seize opportunities, as well as overcome obstacles; but also, hubris may accentuate motivational power and the conviction that the entrepreneurial venture is in good, capable hands (Bass, 1990; Hiller and Hambrick, 2005).

Hubris determines choices, strategy preferences and dispositions (Jensen and Zajac, 2004). Hubristic individuals seek personal power and use this power to support their excessive image of self and to curtail negative feedback to carry out grandiose projects (Kroll et al., 2000; Glad, 2002). Accordingly, hubris may provide entrepreneurs with the bravado to pursue challenging tasks (Hayward et al., 2006; Haynes et al., 2015) and the conviction that they will have the necessary resources for their ventures to succeed (Cialdini, 1993; Lowe and Ziedonis, 2006). Hubristic entrepreneurs might overstate the value and efficacy of their unique personality and leadership skills and, therefore, overestimate the likelihood that their ventures will succeed. Together, these factors illustrate why hubristic entrepreneurs might misjudge gains from prospective ventures and present themselves as greedy (Hayward and Hambrick, 1997; Haynes et al., 2015).

Previous literature has shown that the mechanisms by which growth aspirations are supported rely on individuals’ convictions on themselves (e.g., Davidsson, 1989, 1991). Further, research suggests that inflated estimations of personal abilities to produce success promote higher goals (e.g., Hayward and Hambrick, 1997; Hiller and Hambrick, 2005). Therefore, to the extent that individuals overvalue their knowledge and skills, hubris may appear as one of the basic elements from which growth aspirations are anchored. Therefore:

H1: Hubristic entrepreneurs are more likely to have higher growth aspirations

Entrepreneurs normally act on what they see or, perhaps more importantly, what they think they see (Busenitz and Lau, 1997). There are several reasons why biases might permeate entrepreneurial decisions, including information overload and velocity, high uncertainty, lack of historical information, and organizational routines and time pressure (Busenitz and Barney, 1997; Baron, 2004). The core argument of this strand of the literature suggests that by relying on these biases, entrepreneurs’ are more comfortable making decisions in contexts of ambiguity, uncertainty, and complexity (Simon et al., 2000; Zacharakis and Shepherd, 2001; Busenitz et al., 2003; Holcomb et al., 2009). Thus, entrepreneurs’ perceptions of reality are critical to their subsequent strategic business decisions (Forlani and Mullins, 2000; Krueger, 1993; Krueger and Brazeal, 1994).

The strategic decisions made by entrepreneurs, such as innovative choices (Chrisman and Patel, 2012; De Massis et al., 2013; Classen et al., 2014), are frequently governed by non-economic goals (Baron et al., 2011; Manzaneque et al., 2020); where according to McMullen and Shepherd (2006), one of its prime drivers is the entrepreneur’s motivations. In this sense, in seeking to improve their venture’s organizational performance, entrepreneurs may be likely to pursue innovations (Schoonhoven et al., 1990; Chaney and Devinney, 1992; Roberts, 1999). Regardless of the internal features of the business, such as its absorptive capacity, entrepreneurs with high growth aspirations can see innovation as the most reasonable avenue to achieve the highest possible business growth (Poblete, 2018).

Upon introducing new products, services, business processes, and/or novel business models, it is impossible to accurately foresee the outcomes of the decisions made (Douglas, 2017). Yet, prior studies highlight that including innovative processes is critical for increasing a firm’s performance (Gundry and Welsch, 2001; Cruz-Cázares et al., 2013; Gao and Chou, 2015). Accordingly, innovative choices may naturally appear to be both feasible and desirable in the eyes of entrepreneurs with high growth aspirations (Chrisman and Patel, 2012; De Massis et al., 2013; Classen et al., 2014). Hence, aspiration levels would affect a firm’s outcomes, including its overall business strategy (Lant, 1992; Miller and Chen, 1994; Audia et al., 2000; Gundry and Welsch, 2001) or decisions regarding R&D investments (Bolton, 1993). These, in turn, are expected to increase the likelihood of developing innovations that enhance the growth rate of the business (Greve, 2003; Chen, 2008; Giachetti and Lampel, 2010; Vissa et al., 2010).

Through this motivational process, entrepreneurs may be more prone to acquire new knowledge to augment the business’ absorptive capacity and improve its flexibility and innovation capability (Kim, 1998; Chaudhuri and Tabrizi, 1999). Such capabilities are linked with innovation efficiency and, ultimately, firm performance (Gundry and Welsch, 2001; Zahra and George, 2002; Manzaneque et al., 2020). In this sense, high growth aspirations may act as a motivational driver encouraging entrepreneurs to increase their efforts to run innovations. Therefore:

H2: The greater the aspiration for growth, the greater the propensity to exhibit innovative behaviors

As previously mentioned, while traditional entrepreneurial activity is statistically more likely to fail, and initial plans for a venture provide a weak predictor of future business performance, entrepreneurs are generally confident of their chances to succeed (Coelho, 2010). Under these circumstances, novice entrepreneurs seem to be primarily driven by their interpretation or construal of their experiences (Griffin and Ross, 1991; Kruger and Dunning, 1999). This is largely unaffected by the experiences of others (Ball et al., 1991) or the context of the situation (Moore and Kim, 2003), even when consideration of others’ experiences and situational features could help improve decision accuracy (Hayward et al., 2006).

Prior evidence has observed that novice entrepreneurs are particularly likely to be overconfident in their skills and that their predictions about the future are optimistic (Poblete et al., 2019). Entrepreneurs with limited experience in their current business may underestimate the difficulty of solving more complex problems and so become overoptimistic in their ability to solve them (Weinstein, 1987; Ucbasaran et al., 2007). Thus, novice entrepreneurs may be prone to overestimate, for example, their customers’ understanding of and appreciation of their products (Camerer and Lovallo, 1999).

According to Akstinaite and Sadler-Smith (2021) feelings of excessive pride and arrogance might reduce as entrepreneurs become cognizant of their knowledge gaps (Shepherd et al., 2000). Thus, while entrepreneurs run their business, other business-related entrepreneurs and stakeholders may act as influential models by providing examples of behavior to observe and even imitate. The more time entrepreneurs are involved in their venture, the more time they have to absorb these representing models and re-encode their cognitions (Forbes, 2005). As a result, as entrepreneurs gain experience and engage with others, they can adjust their mental frames based on the behavior they have observed. In this regard, studies have noted that entrepreneurs are more likely to imitate behavior modeled by business-related entrepreneurs they perceive as similar to them or who operate in the same industry (Holcomb et al., 2009).

As they acquire more experience, entrepreneurs moderate their optimism and become more accurate and precise (Ucbasaran et al., 2007; Trevelyan, 2008). This may suggest that the effect of hubris is not static. On the contrary, mental representations are repeatedly configured and calibrated while the entrepreneur is running their business. Indeed, this improved information processing has been evidenced on changes in cognitive structures themselves (Mitchell et al., 2002). Thus, in comparative terms, while novice entrepreneurs may overstate the extent to which their cognitive resources can confer them with a competitive advantage, more experienced entrepreneurs are less likely to overlook new information that contradicts their expectations about their resources and capabilities (Baron and Henry, 2010). Therefore:

H3: The level of entrepreneurial experience moderates the relationship between hubris and growth aspirations, such that this relationship is weaker.

As the relationship between hubris and growth aspirations, there are also theoretical and empirical grounds for suggesting that the level of entrepreneurial experience may play a key role in the relationship between growth aspirations and innovative endeavors. Numerous studies have observed that firms’ age and innovation are negatively related (Hansen, 1992; Huergo and Jaumandreu, 2004; Coad et al., 2016). While there may be several reasons behind this inverse relationship, entrepreneurs’ motivation for growing in established firms is merely one interrelated factor with several other crucial determinants of firm growth (Wang et al., 2016). Zhou and de Wit (2009) demonstrate that several conditions must be in place for a venture to grow, including individual determinants, organizational determinants (firm attributes, firm strategies, firm-specific resources, organizational structure, and dynamic capability), environmental determinants, and growth barriers (financial and institutional barriers). Consequently, the direct influence of the entrepreneurs’ motivation in the outcomes of a firm, such as running innovations, decreases as firms grow.

Further, the influence of entrepreneurial motivation may decrease as the business venture becomes more established. Decisions made by established entrepreneurs might be more pragmatic than those made by their amateur, overly optimistic, counterparts who are at the early stages of the venture creation process and may “want it all.” Amateur entrepreneurs might ignore, for example, the influence of external environmental conditions on growth objectives (Dutta and Thornhill, 2008). In this sense, innovation is no longer seen as an all-powerful force (Poblete, 2018), but only as one of the many roads to achieve growth (Carreón-Gutiérrez and Saiz-Álvarez, 2019). According to Gundry and Welsch (2001), high-growth–oriented entrepreneurs tend to adopt a more structured approach to organizing their businesses, characterized by: strategic intentions that emphasize market growth and technological change, a stronger commitment to the success of the business, greater willingness to sacrifice on behalf of the business, earlier planning for the growth of the business, utilization of a team-based organizational design, concern for reputation and quality, adequate capitalization, strong leadership, and utilization of a wider range of financing sources for the expansion of the venture (p. 454). Balancing these elements with entrepreneurs’ notions of different growth strategies might reduce the influence of motivation on innovative endeavors.

Davidsson (1991) found that perceived ability, perceived need, and perceived opportunity are some of the most important influences on growth motivation.^1^ Hence, this growth bias, expressed by innovation-driven “highly ambitious” entrepreneurs, is more likely to be evidenced as an early growth plan in the life of a business and actioned by novice entrepreneurs (Davidsson, 1991; Storey, 2011; Uy et al., 2013). Accordingly, as firms become more established, entrepreneurs become less “entrepreneurial” and not aggressively growth-oriented through innovation. As entrepreneurs progress through the entrepreneurial process (Bhave, 1994; Trevelyan, 2008), they acquire experiential knowledge and a heightened awareness of external environmental conditions. This knowledge provides a frame of reference through which entrepreneurs’ mental images of innovations are actively shaped by the entrepreneur’s sense-making processes (Autere and Autio, 2000), which reduce the link between innovation and growth orientations. Therefore:

H4: Level of entrepreneurial experience moderates the relationship between growth aspirations and innovative behaviors, such that this relationship is weaker

**FIGURE 1:**
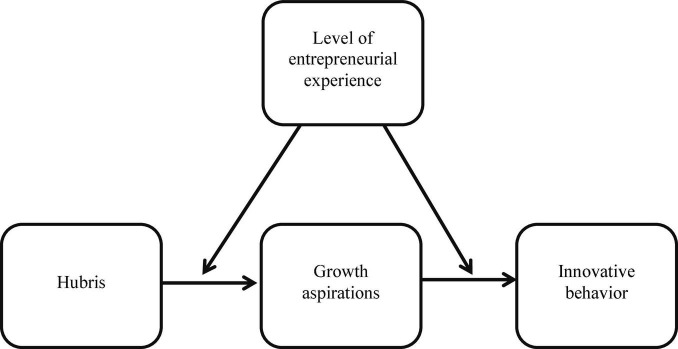
A moderated mediation model of the influence of hubris on innovations.

In sum, this study proposes, and tests, a moderated mediation model of the role of growth aspirations on innovative behaviors. This model (represented in Figure 1) proposes that among entrepreneurs, hubris stimulates growth aspirations and that these ambitions, in turn, promote innovative endeavors. Further, the model also suggests that both of these links are moderated by the entrepreneurial phases, becoming weaker as entrepreneurs advance through more established stages of the venture.

## Methodology

### Sample

The data used for the analysis originate from the 2015 to 2017 Adult Population Surveys of the Global Entrepreneurship Monitor (GEM) project (Reynolds et al., 2005). Pooling the observations from three consecutive years in one dataset allows for controlling for fluctuations in the distribution of entrepreneurial innovativeness across countries and time. GEM is currently the largest and most widely recognized cross-country research initiative to study the prevalence, determinants, and consequences of entrepreneurial activity. The core activity of GEM is to compile yearly empirical data on entrepreneurial activity based on a random sample of at least 2,000 adult-age individuals in each of the participating countries.

### Variables

#### Hubris

According to Hayward et al. (2006), hubris’ conceptuality relates to an individual’s subjective interpretation of information concerning three separate and independent psychological processes: (1) overconfidence in knowledge, (2) overconfidence in predictions, and (3) overconfidence in personal abilities. GEM data also provide relevant information relating to these dimensions. To proxy for overconfidence in knowledge, this study uses individuals’ beliefs of their skills, knowledge, and experience to successfully run a startup. The entrepreneur’s view that in the next 6 months good business opportunities will arise is employed to measure overconfidence in predictions. Finally, overconfidence in personal abilities is measured by the belief that in the next 5 years, the startup will hire 19 workers or more.

The rationale for selecting the above-defined categories is as follows: The primary objective is to differentiate between overconfident entrepreneurs and those who exhibit some degree of confidence. Accordingly, the strictest possible definition for hubris that the data allows is defined as the reference category. Thus, and considering a robust finding in cognitive psychology highlighting that high confidence is positively correlated with greater overconfidence (Hayward et al., 2006), hubristic entrepreneurs are defined as those whose answers meet all three categories. Every other combination is zero.

#### Growth Aspirations

Similarly, like other studies, growth aspirations are calculated as the difference between the natural logarithm of entrepreneurs’ expected number of employees in the next 5 years and the actual number of employees at inception (Estrin et al., 2013; Capelleras et al., 2019).

#### Innovative Behavior

In concordance with previous literature (e.g. Poblete, 2018; Carreón-Gutiérrez and Saiz-Álvarez, 2019), this study uses the presence of innovative outcomes at a firm-level to proxy for innovative behavior. The underlying argument about this logic relies on the notion that the presence of firm-level innovative outcomes may emerge as a consequence of initial behavioral responses that individuals perform and engage (Shaver, 2003).

The GEM survey includes three follow-up questions relating to the innovativeness of the business idea of those individuals who qualify as entrepreneurs. These questions cover various aspects of the business venture, including the novelty of the technology, the novelty of the product or service for customers, and the degree of market competition. They are widely used to construct a profile of the innovativeness of business ventures (Poblete, 2018; Carreón-Gutiérrez and Saiz-Álvarez, 2019). They also allow defining various measures for the types and degrees of entrepreneurial innovativeness.

Recognizing that innovations can emerge at different stages of the entrepreneurial venture and in various forms, this study covers some of the potential manifestations that suggest the presence of innovation. Concretely, innovation is likely to arise in settings characterized by: few or no direct competitors; novelty in products and services for customers; and high use of technology. More evidence of these manifestations means that it is more likely that the venture is innovative. The variable ranges from 1 to 6.

#### Entrepreneurial Experience

Following the trail of previous research (e.g., Poblete, 2018; Poblete et al., 2019), entrepreneurial phases are used as a proxy for the level of entrepreneurial experience. Entrepreneurial activity is often categorized by identifying the different phases of the new venture development process (Gatewood et al., 1995; Shook et al., 2003; Brockner et al., 2004; Forbes, 2005; Baron and Shane, 2007). GEM defines the entrepreneurial process based on three transition points marking typical entrepreneurial barriers (Reynolds et al., 2005). The first phase of the start-up process consists of people in the adult population contemplating setting up a business (Shaver et al., 2001). During this phase, entrepreneurs move from conception to gestation or start-up process and are considered nascent entrepreneurs.

The second transition reflects the development of the start-up into an operational business: the firm’s birth transition (Choi et al., 2008). Many aspects can be part of the new firm’s “birth” (Katz and Gartner, 1988; Reynolds and Miller, 1992), such as the intention to create a business, boundary-type definitions, resource-based definitions, and/or definitions motivated by exchanges. GEM, however, defines new business owners as those entrepreneurs that have paid salaries and wages for more than 3 months and less than 3.5 years (Reynolds et al., 2005).

The third phase has to do with the liability of newness and overcoming the valley of death. This final stage is reflected by the age of the firm. Thus, those entrepreneurs that have paid salaries and wages.

#### Control Variables

A total of 12 control variables are included. Three are country-level variables: Global Competitiveness Index (GCI), country dummies, and year dummies. Two are firm-specific variables: industry-sector dummies and export intensity. Finally, seven variables relate to the entrepreneur: gender, age, educational level, social capital, fear to fail, entrepreneurial intentions, and whether he/she has prior entrepreneurial experience. Table 1 presents a detailed description of the control variables.

**TABLE 1:** Description of the control variables.

Variable	Description/Survey question	Answer categories
Gender	What is your gender?	Male: 1; Female: 0
Age	What is your current age (in years)?	1–99
Educational level	Educational attainment	Pre-primary education: 1; Primary education or first stage of basic education: 2; Lower secondary or second stage of basic education: 3; (Upper) secondary education: 4; Post-secondary non-tertiary education: 5; First stage of tertiary education: 6; Second stage of tertiary education: 7
Social capital	Do you know someone personally who started a business in the past 2 years?	Yes: 1; No: 0
Fear to fail	Would fear of failure prevent you from starting a business?	Yes: 1; No: 0
Ent. Intentions	Are you, alone or with others, expecting to start a new business, including any type of self-employment, within the next 3 years?	Yes: 1; No: 0
Prior entrep. Experience	Have you, in the past 12 months, sold, shut down, discontinued or quit a business you owned and managed, any form of self-employment, or selling goods or services to anyone?	Yes: 1; No: 0
Export intensity	What percentage of your annual sales revenues will usually come from customers living outside your country?	0–100%
GCR–5 categories	Global Competitiveness Report, Country Group report–5 categories	Stage 1: Factor-driven; Stage 2: transition to stage 3; Stage 3: Efficiency-driven; Stage 4: transition to stage 5; Stage 5: Innovation-driven

These variables are selected based on previous studies showing that these characteristics play an important role in influencing entrepreneurial decisions (Koellinger et al., 2007; Parker, 2009). For example, fear of failure inhibits innovations, both by suppressing new ideas and avoiding risky concepts (Kuyatt, 2011). Further, studies suggest that being a male enhances growth aspirations (Estrin and Mickiewicz, 2011) and innovative behavior (Koellinger, 2008). In addition, age has been considered as a factor affecting entrepreneurial growth aspiration (Autio, 2005) and also strategic decisions of the firm, including innovative choices (Busenitz and Barney, 1997). Further, controlling by industry accounts for sectoral differences in capital intensity ratio and optimum size of the firm that may influence growth aspirations (Estrin et al., 2013) and propensity to innovation (Chaney and Devinney, 1992; Bolton, 1993; Cruz-Cázares et al., 2013). Similarly, by adding the GCI, this study controls for different aspects of country-level competitiveness, such as the level of innovation and business sophistication (Amorós et al., 2019).

## Results

Table 2 summarizes the means, standard deviations, and correlations for all variables. Tables 3, 4 present the results of the analyses performed to examine the moderation effect of hubris and the entrepreneurial process on growth aspirations. Table 4 presents the results of the corresponding analysis for innovative behavior.

**TABLE 2:** Mean, standard deviation, and correlations.

	Variables	Mean	*SD*	1	2	3	4	5	6	7	8	9	10	11	12
1	Gender	1.410	0.492												
2	Age	41.170	12.804	-0.020**											
3	Educational level	3.231	1.490	-0.048**	-0.044**										
4	Social capital	0.609	0.488	-0.030**	-0.126**	0.087**									
5	Fear to fail	0.299	0.458	0.052**	0.002	-0.015**	-0.043**								
6	Ent. Intentions	0.398	0.489	-0.023**	-0.217**	0.000	0.117**	-0.039**							
7	Prior entrep. experience	0.078	0.269	-0.009**	-0.038**	0.003	0.051**	0.016**	0.147**						
8	Export intensity	3.228	1.144	0.040**	0.035**	-0.114**	-0.041**	0.005	-0.087**	-0.046**					
9	GCR—5 categories	3.736	1.227	-0.061**	0.198**	0.335**	-0.054**	0.002	-0.222**	-0.067**	-0.128**				
10	Hubris	0.040	0.197	-0.071**	-0.027**	0.096**	0.069**	-0.047**	0.086**	0.033**	-0.069**	0.030**			
11	Entrep. phases	2.116	0.851	-0.038**	0.287**	-0.081**	-0.084**	0.009**	-0.273**	-0.062**	0.115**	0.038**	-0.018**		
12	Growth aspirations	1.460	1.248	-0.138**	-0.042**	0.187**	0.054**	-0.036**	0.124**	0.064**	-0.154**	0.075**	0.494**	-0.097**	
13	Innovative behavior	5.772	1.499	-0.026**	0.042**	0.059**	0.025**	-0.048**	0.080**	0.016**	-0.042**	0.050**	0.082**	0.051**	0.131**

*p < 0.010; **p < 0.005; ***p < 0.001.

**TABLE 3:** Results of the moderation effect of hubris and entrepreneurial process on growth aspirations.

Variables	Growth aspirations			
	Model 1	Model 2	Model 3	Model 4
Country (dummies)	Yes	Yes	Yes	Yes
Year (dummies)	Yes	Yes	Yes	Yes
Gender	-0.262***	-0.187***	-0.198***	-0.198***
Age	-0.003***	-0.004***	-0.002***	-0.002***
Educational level	0.132***	0.097***	0.092***	0.093***
Social capital	0.076***	0.008	0.012	0.012
Fear to fail	-0.075***	-0.021*	-0.025**	-0.025**
Ent. Intentions	0.291***	0.229***	0.188***	0.189***
Prior entrep. experience	0.2***	0.158***	0.152***	0.152***
Export intensity	-0.131***	-0.101***	-0.093***	-0.094***
Industry (1)	-0.146***	-0.086**	-0.057*	-0.06*
Industry (2)	0.005	0.012	0.042	0.041
Industry (3)	-0.048	-0.036	-0.005	-0.006
Industry (4)	-0.293***	-0.203***	-0.183***	-0.184***
GCR—5 categories	0.001	-0.004	-0.01**	-0.01**
Hubris		2.11***	2.137***	2.294***
Entrep.Phases (E.P.)			-0.137***	-0.131***
E.P.* Hubris				-0.08***
*R* ^2^	0.099	0.301	0.308	0.309
Adjusted *R*^2^	0.099	0.301	0.308	0.309
Change in *R*^2^		0.202	0.007	0.001

*p < 0.010; **p < 0.005; ***p < 0.001.

**TABLE 4:** Results of the moderated mediation analysis.

Variables	Innovative behavior					
	Model 1	Model 2	Model 3	Model 4	Model 5	Model 6
Country (dummies)	Yes	Yes	Yes	Yes	Yes	Yes
Year (dummies)	Yes	Yes	Yes	Yes	Yes	Yes
Gender	-0.064***	-0.053***	-0.044***	-0.045***	-0.026	-0.031*
Age	0.007***	0.007***	0.005***	0.005***	0.007***	0.008***
Educational level	0.039***	0.034***	0.037***	0.037***	0.017***	0.017***
Social capital	0.052***	0.041***	0.047***	0.045***	0.016	0.010
Fear to fail	-0.144***	-0.135***	-0.136***	-0.136***	-0.142***	-0.138***
Ent. Intentions	0.281***	0.265***	0.316***	0.312***	0.262***	0.258***
Prior entrep. exp	0.043**	0.035**	0.044**	0.042**	0.023	0.013
Export intensity	-0.036***	-0.032***	-0.040***	-0.038***	-0.018***	-0.018***
Industry (1)	-0.172***	-0.167***	-0.202***	-0.195***	-0.221***	-0.206***
Industry (2)	-0.044	-0.046	-0.078***	-0.075***	-0.105**	-0.101**
Industry (3)	-0.012	-0.012	-0.042	-0.038	-0.002	0.008
Industry (4)	0.046*	0.055**	0.033	0.036	0.136***	0.138***
GCR - 5 categories	0.042***	0.041***	0.044***	0.044***	0.078***	0.078***
Hubris		0.517***	0.507***	-0.418***	-0.761***	-0.006
Entrep.Phases (E.P.)			-0.137***	-0.117***	-0.324***	-0.105***
E.P. * Hubris				0.454***	0.355***	-0.043
Growth aspirations (G.A.)					0.194***	0.135***
E.P* G.A.						-0.175***
*R* ^2^	0.019	0.024	0.029	0.032	0.064	0.073
Adjusted *R*^2^	0.019	0.024	0.029	0.031	0.064	0.072
Change in *R*^2^		0.005	0.005	0.002	0.033	0.009

*p < 0.010; **p < 0.005; ***p < 0.001.

Hypothesis 1 predicts that entrepreneurs’ hubris is positively related with their growth aspirations. Results relevant to this hypothesis are presented in Model 2 of Table 3 and indicate that, as predicted, hubris is significantly related to growth aspirations (*B* = 2.11, *p* < 0.001). Hypothesis 2 predicts that growth aspirations are positively related to the presence of innovative endeavors. Results (Model 5 of Table 4) provide support for H2: growth aspiration is significantly related to an innovative behavior (*B* = 0.194, *p* < 0.001).

Hypothesis 3 proposes a moderating effect of the level of entrepreneurial experience on the relationship between hubris and growth aspirations. As shown in Model 4 of Table 3, results offer support for Hypothesis 3 (B of the interaction between hubris and growth aspirations = –0.08, *p* < 0.001). Consistent with predictions, the link between hubris and growth aspirations is indeed weaker for entrepreneurs that have been running their venture for longer.

Hypothesis 4 predicts a moderating role of the level of entrepreneurial experience with respect to the relationship between growth aspirations and innovative behaviors. Results offer support for H4 (Model 6 of Table 4): the level of entrepreneurial experience negatively moderates the relationship between entrepreneurs’ growth aspirations and different manifestations of innovative entrepreneurial activity (B of the interaction between value of innovation and hubris = –0.175, *p* < 0.001).

### Mediating Role of Growth Aspirations

To test the proposal that the experience-moderated growth aspirations mediate the relationship between hubris and innovative behavior, we follow the procedure by Baron and Kenny (1986). It poses that the following conditions must be met: (a) the independent variable is a significant predictor of the mediator, (b) the mediated variable is a significant predictor of the dependent variable in the absence of the mediator, (c) the mediator has a significant unique effect as a predictor of the dependent variable, and (d) the effects of the independent variable on the dependent variable shrinks upon the addition of the mediator to the model. Full mediation is indicated if the effect of the independent variable is no longer significant when the mediating variable is added, whereas partial mediation is suggested if the effect of the independent variable is reduced but remains significant.

First, it was examined the relationship between the independent variable and the mediator. As shown in Table 3, a significant relationship exists between experience-moderated hubris and growth aspirations (*B* = –0.08, *p* < 0.001). Second, looking at the Model 4 of Table 4, which presents the relationship between the independent variable and dependent variable, it is possible to observe that experience-moderated hubris is significantly related to innovative behaviors (*B* = –0.454, *p* < 0.001). Third, experience-moderated growth aspirations is significantly associated with innovative behaviors (*B* = –0.175, *p* < 0.001) as indicated in Model 6 of Table 4. Fourth and finally, as Models 4 and 6 in Table 4 demonstrate, the coefficient for the experience-moderated effects of hubris on innovative behaviors became insignificant when the experience-moderated effects of growth aspirations were included in the regression equation. The coefficient decreased from .454 (*p* < 0.001 in Model 4) to -0.043 (n.s. in Model 6). Thus, experience-moderated growth aspirations fully mediate the positive relationship between experience-moderated hubris and innovative behaviors in the present data.

Sobel tests were used to obtain further evidence of full mediation. Sobel (1982) tests calculate the magnitude of the unstandardized indirect effect and its associated standard error. The ratio of the indirect effect over its standard error is referred to as the Sobel statistic, which is compared to a z distribution to determine the statistical significance of the indirect effect. The Sobel tests showed that the indirect effect of experience-moderated hubris on innovative behaviors (Sobel statistic = –13.95, *p* < 0.001) was in the anticipated direction and statistically significant, providing further evidence for full mediation.

## Discussion

This study investigated the relationships between hubris, growth aspirations, and innovative behavior. Both hubris and growth aspirations were chosen based on the extensive evidence indicating their relevance for behaviors performed by entrepreneurs. Further, growth aspirations have often been identified as an essential antecedent of innovation, while hubris has been found to influence many aspects of cognition and behavior. Based on such evidence, it has recently been suggested that hubris may also play an important role in entrepreneurship, influencing entrepreneurs’ decisions to pursue product newness, low competition, and recent technology.

The findings provide empirical evidence for these arguments. Consistent with a large body of evidence, the results indicate that entrepreneurs’ hubris is significantly related to their growth aspirations. Further, enhanced growth aspirations were found to be significantly related to the development of ventures presenting innovative outcomes. Overall, these findings indicate that hubris relates to important aspects of entrepreneurship—a finding consistent with results recently reported in the entrepreneurship literature. However, such effects are not direct in nature; rather, they are mediated by intervening variables. Specifically, in the present research, growth aspirations were found to mediate the relationship between entrepreneurs’ hubris and innovative behavior.

The results indicate that both the relationship between hubris and growth aspirations, and between growth aspirations and innovation, are moderated by the entrepreneurial phases in which the entrepreneur is involved. Both relationships are weaker in the advanced rather than the early entrepreneurial phases. Although this study does not aim to explain the mechanisms underlying these moderating effects, both are predicted based on previous research. Concerning the link between hubris and growth aspirations, it was reasoned that advanced entrepreneurial phases are less likely to present over-optimism, a condition found in recent studies to be required for hubris to enhance growth aspirations. Turning to the relationship between growth aspirations and innovative endeavors, it was reasoned that innovation is easier for new firms than for established firms and consequently, the impulses generated by the entrepreneurs’ aspirations are more likely to be carefully considered and implemented in established firms than new ventures. Thus, the relationship between growth aspirations and innovative behaviors will be weaker as firms age.

## Implications

This study makes three important contributions. First, it studies the entrepreneurial process using an agentic approach. This allows exploring and further understanding how “entrepreneurial rosy-lenses” evolve across the different phases of an entrepreneurial venture (Douglas, 2009). Through hubris, entrepreneurs set ambitious goals and decide on the courses of action that are most likely to produce the desired outcomes (Bandura, 1977). In this sense, hubris operates as a powerful intrinsic motivator for amateur entrepreneurs and as a core guide for their actions. As entrepreneurs progress to more experienced phases of the business, they acquire a forethoughtful perspective calibrated to plan ahead, reorder their priorities, and restructure their strategies. Thus, business decisions in more advanced stages seem to be complemented, providing direction, coherence, and meaning to the business.

Secondly, this study combines two theoretical approaches: one purely borrowed from psychology and other centered on entrepreneurship.^2^ This provides a broad overview to effectively explain how, why, and when hubris can be conceived as one of the antecedents of innovative endeavors. In other words, linking these theories offers a more inclusive explanation for a phenomenon observed in many studies (Shane, 2008, 2009). Additionally, it provides more empirical legitimacy through the data and analyses performed in this study. Entrepreneurship is a dynamic and developmental process that culminates with the exploitation of a business opportunity (Davidsson, 2015). This research reveals that motivations and aspirations are not fixed, but rather grounded in the different stages through which entrepreneurs evolve. While extensive research has developed the notion of the study of bias in entrepreneurship (Zhang and Cueto, 2017); this study likewise saw the possibility for additional research on integrative approaches. Thus, it responds to calls for socially situated cognition research (Mitchell et al., 2011; Randolph-Seng et al., 2015; Davidsson, 2016).

Third, the findings offer valuable insights for policymakers on the nexus between innovative endeavors and hubristic entrepreneurship. Prior studies have shown that many entrepreneurs who participate in training and education programs only realize what it means to be an entrepreneur during the course of the activity and end up adjusting their aspirations (Oosterbeek et al., 2010). Consequently, public policies that are tightly focused on removing entry barriers for prospective entrepreneurs may result in a large pool of entrepreneurs with inadequate entrepreneurial skills (Camerer and Lovallo, 1999; Cassar, 2010) potentially leading to more harm than good (Anokhin and Wincent, 2012). It is important to note that this study does not suggest that promoting innovation is detrimental to entrepreneurial activity. It warns, however, that innovative endeavors should not be encouraged for the wrong reasons, especially for novice entrepreneurs. This is crucial to prevent ineffective entrepreneurship policies which may lead to undesired outcomes.

Finally, I further argue that the relationship between hubris and innovative behavior is partly indirect since it is mediated by growth aspirations where the entrepreneurial experience has a relevant moderating role. Novice entrepreneurs are more likely than experienced entrepreneurs to be influenced by their hubris in shaping their growth aspirations, and these highly aspirational inexperienced entrepreneurs, in turn, are particularly prone to behave in an innovative manner. Thus, the combined effects of growth aspirations with the lack of entrepreneurial experience from which hubris operate in the mind of naive entrepreneurs seem to confer some insights about why a relevant percentage of innovative entrepreneurial activity fail in the early stages of the firms (Shane, 2008; Zhang and Cueto, 2017). Presumably, experienced entrepreneurs constrain their growth bias through innovation as they develop a more accurate understanding of their knowledge, skills, and abilities; while also exploring different growth strategies (Uy et al., 2013; Mueller and Shepherd, 2016). As a result, the present research sheds light on the factors that contribute to being aware of the potential downsides of innovation in the hands of novice hubristic entrepreneurs highly motivated to grow (Davidsson, 1991; Storey, 2011). In terms of managerial implication, it is recommended that innovative strategies may be selected on the advanced stages of the venture to the extent that entrepreneurs’ growth aspirations are not blinded by hubris.

## Limitations and Suggestions for Future Research

While the results of this study are in line with the findings of several previous studies, limitations exist that should be carefully addressed by future research. For instance, many of the measures employed are self-reported (e.g., hubris, growth aspirations, innovation). Also, among the limitations of the present work is the difficulty of directly measuring constructs used and thus the need to use “proxy” variables. Although these measures were based on those used in previous studies and possess acceptable reliability and academic validity, the constructs of primary interest are complex. Thus, additional measures of these variables should be incorporated before the results of this research can be accepted with confidence.

Secondly, this study did not examine other potential mediators of the relationship between hubris and innovation aside from growth aspirations. Although results indicate that growth aspirations fully mediate the relationship between hubris and innovation, hubris may also influence innovation through other mechanisms not specifically investigated here. Future research should explore the role of other potential mediators of the relationships between hubris and firm-level variables such as innovation.

Future studies could address whether the Dunning-Krueger effect is evidenced among entrepreneurs. In broad terms, the Dunning-Krueger effect is the finding that across a wide range of tasks, poor performers greatly overestimate their ability, whereas top performers make more accurate self-assessments. While the findings of this research provide some insights that may suggest that novice entrepreneurs are indeed prone to overvalue their human capital (knowledge, abilities and skills), there is limited information about expert entrepreneurs in regard to how much they value their entrepreneurial self-efficacy, for example.

There is also a need to examine the potential role of hubris in other aspects of the entrepreneurial process. For instance, does hubris play a role in discovering emerging opportunities? Countries like Chile, which have been encouraging entrepreneurship and increasing their entrepreneurial activity rates, may be studied under the lens of normative social influence. Social psychology research also suggests that individuals are prone to optimistic bias. This bias induces people to believe that they are above the average in several domains. Hubris can increase this bias by enhancing an entrepreneur’s capacity to discover new opportunities. In turn, investigating the role of conformity (individuals acting per the societal rules) could provide valuable insights into the nature of the foundations in the HTE. Based on prior literature suggesting that social norms have a significant effect on opportunity confidence (Hopp and Stephan, 2012; Emami and Khajeheian, 2018), normative social influence can be an antecedent of why so many people decide to enter in entrepreneurship.

Finally, future research should examine the expert knowledge in entrepreneurship and how to cultivate it. Previous research suggests that the entrepreneurial mindset is malleable and trainable, so deeper investigation of the transition from novice cognitive structures to expert cognitive structures would appear to be an important and potentially valuable task for entrepreneurial education.

## Conclusion

The results of this study add to the existing body of knowledge investigating the role of hubris in innovative entrepreneurship, showing that entrepreneurs’ hubris is, directly and indirectly, related to innovation. In a general sense, it suggests that hubris can influence entrepreneurs’ levels of growth aspirations. However, this effect is stronger when entrepreneurs are at the initial stages of the entrepreneurial process (e.g., novice entrepreneurs). As they progress through the different phases of the process, the influence of hubris on their growth aspirations decreases. Similarly, growth aspirations encourage innovative behaviors, but as entrepreneurs advance through the process, further increments in growth aspirations are associated with declines, rather than advances, in innovative behaviors. Novice entrepreneurs must become cognizant of the risks that hubris can confer and regulate it accordingly—particularly at the early stages of their entrepreneurial venture. They must also recognize that innovations are not necessarily the best and only mean to reach business growth.

## Data Availability Statement

Publicly available datasets were analyzed in this study. This data can be found here: www.gemconsortium.org.

## Author Contributions

CP was responsible for all the work done in this study.

## Conflict of Interest

The author declares that the research was conducted in the absence of any commercial or financial relationships that could be construed as a potential conflict of interest.

## Publisher’s Note

All claims expressed in this article are solely those of the authors and do not necessarily represent those of their affiliated organizations, or those of the publisher, the editors and the reviewers. Any product that may be evaluated in this article, or claim that may be made by its manufacturer, is not guaranteed or endorsed by the publisher.

## Footnotes

1 This growth motivation in turn can be expressed in several ways, such as aspirations, expectations, and behavioral intentions, among others (Davidsson, 1989).

2 However, the concept of “hubris” has been studied previously in both psychology and management.
